# An Assessment of the Motor Performance Skills of Children with Autism Spectrum Disorder in the Gulf Region

**DOI:** 10.3390/brainsci10090607

**Published:** 2020-09-03

**Authors:** Rehab H. Alsaedi

**Affiliations:** 1 Faculty of Education, Queensland University of Technology (QUT), Brisbane 4059, Australia; r-alsaidi@hotmail.com; 2 Department of Special Education, Taibah University, Madinah 41477, Saudi Arabia

**Keywords:** motor performance skills, autism spectrum disorder, Gulf, BOT-2

## Abstract

This study aims to determine the prevalence, severity, and nature of the motor abnormalities seen in children with autism spectrum disorder (ASD) as well as to elucidate the associated developmental profiles. The short-form of the Bruininks-Oseretsky Test of Motor Proficiency, Second Edition (BOT-2) was used to assess various aspects of the motor performance of 119 children with ASD and 30 typically developing children (age range: 6–12 years) from three Gulf states. The results revealed the high prevalence of motor abnormalities among the ASD group when compared with the normative data derived from the BOT-2 manual as well as with the data concerning the typically developing group. The results also indicated that the motor performance of the children with ASD fell within the below-average range according to the BOT-2 cut-off score. Further, the results suggested that the age variable may influence the overall motor performance of children with ASD, since the children’s motor abnormalities may decrease with maturation. The results concerning the specific motor dysfunction profiles seen in individuals with ASD could help practitioners, parents, and educators to better understand the nature of the motor deficits exhibited by children with ASD, which could assist with the design and implementation of treatment and rehabilitation programs for such children. Overall, motor performance represents an important aspect that should be considered during the clinical evaluation of ASD and that should not be ignored during early interventions.

## 1. Introduction

Motor skills are associated with “activities that require a chain of sensory (vision, hearing, touch, and smell), central (brain and nervous systems), and motor mechanisms whereby the performer is able to maintain constant control of the sensory input and in accordance with the goal of the movement” [1]. The child development literature tends to assume that motor ability is an important indicator of overall development, particularly if it is compromised [2]. Indeed, early motor disturbances and delays are likely to have far-reaching consequences for later development, and they may be predictive of developmental disorders in later life [3,4]. Children with autism spectrum disorder (ASD) represent a population likely to experience motor impairments [5].

ASD affects every domain of human existence. An impairment in the motor domain can have profound effects on a child’s development in areas such as schooling, socialisation, and communication [6,7]. It has been estimated that “80–90% of children with ASD show some degree of motor abnormality” [8]. However, it should be noted that previous results regarding prevalence rates vary depending on the utilised cut-off scores, diagnoses, and instruments [9].

Motor dysfunction represents an important neurological symptom in those with ASD. A growing body of evidence illustrates the motor difficulties experienced by children with ASD when compared with their typically developing peers [10,11,12,13]. However, such difficulties have traditionally been considered secondary aspects of ASD [14].

The only motor abnormalities currently included in the diagnostic criteria for ASD are stereotypical and repetitive behaviours [15]. However, the relevant motor abnormalities are not limited to such behaviours, as individuals with ASD, including children [16], often display motor issues consisting of both delays and deficits. Delays occur in the gross and fine motor skills [12], while deficits manifest in praxis [17], coordination and gait [18], postural control [19], and motor planning [20]).

Little is currently known about the underlying neurobiology of ASD-related motor problems, although the disruptions observed in those with ASD may stem from abnormal brain connectivity [10,21]. Some studies report reduced cerebellar activation in individuals with ASD during motor tasks when compared with neurotypical controls [22,23]. Dysfunctions in the frontostriatal pathways of children with ASD have also been demonstrated [24,25]. Further, the aberrant neural connectivity patterns observed between the cerebellar and frontostriatal pathways indicate the role of the cortical and subcortical structures and the pathways connecting them in controlling the accuracy of motor outputs [21].

Considerable ambiguity persists regarding the nature of such motor abnormalities and whether they are universal and specific to ASD. Some findings indicate that motor impairments constitute a cardinal feature of ASD [10,26,27,28]. The identified impairments remain consistent across ages and levels of functioning [11,29,30]. Additionally, evidence from prospective studies of at-risk infants indicates motor deficits to have been documented in infants later diagnosed with ASD [31,32,33,34]. Therefore, motor symptoms could serve as diagnostic markers and guide the early identification of ASD [26,35,36]. However, other evidence suggests that motor delays can be attributed to developmental disorders in general, meaning that they are not specific to ASD [37].

Some preliminary evidence indicates that motor impairments represent an essential part of a broader autistic phenotype [38], although other studies have not found such impairments to represent an essential aspect [8,37]. It is important to recognise that motor impairments are not limited to one disorder (e.g., ASD), as they are also present in other developmental disorders [9,39,40,41]. The debate regarding the prevalence and importance of motor deficits is not surprising given that ASD is a spectrum disorder, meaning that every child exhibits a wide variety of symptoms with different degrees of severity. This variability can offer a unique opportunity to identify disorder subtypes [42].

Jeste (2011) offers a number of justifications for investigating motor dysfunction in individuals with ASD [20]. First, motor symptoms are easily quantifiable and so can be objectively measured. Second, motor impairments may elucidate the heterogeneity within the autistic spectrum. Third, the investigation of motor abnormalities may yield insights into the aberrant neural mechanisms underlying ASD and the defining characteristics of the disorder. Fourth, motor performance is critical to the development of a wide variety of skills, which would all benefit from the early identification of deficits and the implementation of appropriate interventions. Lastly, determining when motor deficits manifest in individuals with ASD may assist with the early diagnosis of the disorder [20].

Age might be a critical factor in determining the nature of the motor dysfunction seen in individuals with ASD. However, the available research concerning the developmental acquisition of motor skills by children with ASD is limited. Evidence suggests that the different subsystems supporting motor development mature at different times and rates, which results in nonlinear development [43]; thereby, indicating the dynamic development of motor skills. Thus, Darrah et al. (2003) suggest the adoption of a developmental change perspective rather than one that emphasises the stability of motor development [44].

No firm conclusions regarding the stability or instability of motor performance can yet be drawn in relation to ASD. Some longitudinal studies indicate the persistent and constant nature of impaired motor performance in children with ASD [45]. Further, other longitudinal research confirms that motor problems among preschool children are not always stable, although they do appear to be so in most children with ASD [46]. However, some cross-sectional studies note the changing nature of motor problems in children with ASD, with the prevalence of motor deficits being lower in older children than younger children [16]. In contrast, other studies indicate that motor difficulties become increasingly problematic over time [34]. A number of factors could contribute to this decline in motor performance, including a lack of early intervention programs targeting motor skills and a lack of parental involvement in children’s treatment [34].

A growing body of evidence gathered in Western contexts suggests motor abnormalities to be a marker of clinical severity and an important therapeutic target in children with ASD [26,36,47]. However, despite this increasing recognition, the evaluation and intervention processes related to motor abnormalities are still not seen as priorities, meaning that they are unlikely to attract the attention of professionals in our research context (the Gulf region). Indeed, motor dysfunctions arguably represent one of the most commonly overlooked comorbidities associated with ASD in the Gulf context. It is important to conduct research regarding motor performance in individuals with ASD in different cultures so as to achieve a clearer understanding of ASD and its phenotypes in different socio-cultural contexts.

The present study had three main aims. First, to determine the ratio of motor impairments seen in children with ASD, as identified using the short form of the Bruininks-Oseretsky Test of Motor Proficiency, Second Edition (BOT-2) [48]. It was expected that children with ASD would surpass the cut-off point for being “motor impaired”. Second, to examine the extent of the differences in the motor performance of children with ASD and typically developing children. It was hypothesised that the motor performance of children with ASD would be significantly worse than that of typically developing children. Third, to investigate the effect of chronological age on the motor performance of children with ASD. Due to the lack of relevant evidence, no predictions were made regarding the relation between motor performance and age.

## 2. Materials and Methods

### 2.1. Study Design

This observational study applied a quantitative research approach that entailed the use of a cross-sectional design to characterize the motor proficiency levels of the participants at a given point in time. There were a number of reasons why this particular research approach was chosen. First, the study relied on a quantitative measurement instrument (i.e., the BOT-2) that provided a numerical evaluation of various aspects of the participants’ motor performance, which meant that it was possible to objectively assess the performance of the study sample. Second, due to the nature of the research aims, outcomes in the form of numerical values were required to allow for comparisons between the ASD population and the corresponding BOT-2 norms and/or those children without ASD, as well as to reflect the relationship between the participants’ age and their test outcomes.

### 2.2. Participants

Data for the present study were collected in Bahrain, Saudi Arabia, and the United Arab Emirates (UAE). The participants comprised children who were participating in an ongoing large-scale study evaluating neurobehavioral problems in children with ASD. They were recruited through purposive sampling on a voluntary basis.

**Figure 1:**
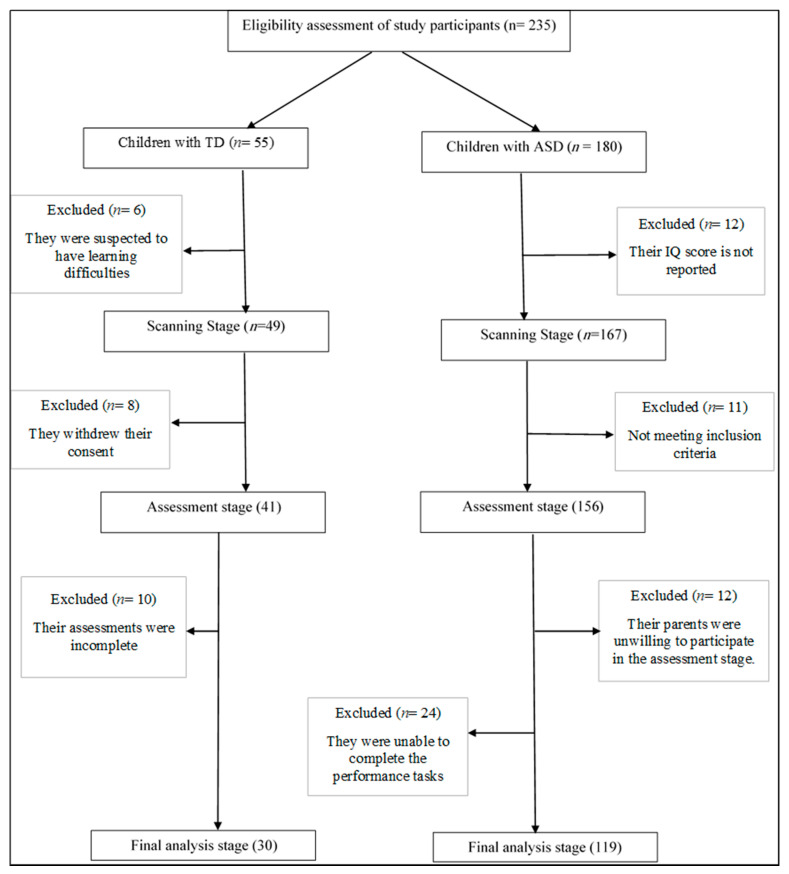
Recruitment of the Participants.

The initial sample consisted of 235 children, who were divided into the ASD (*n* = 180) and typically developing (TD) (*n* = 55) groups. Among them, 61 children with ASD and 25 TD children were excluded from the final sample. The recruitment process is illustrated in Figure 1.

All the children with ASD (age range: 6–12 years; mean age = 8.75 years) had received a formal diagnosis prior to their recruitment for the present study. They attended three different types of educational settings, namely a fully inclusive school (*N* = 26; 21.8%), a partially inclusive school (*N* = 19; 15.9%), or a specialised ASD school or centre (*N* = 74; 62.18%).

The inclusion criteria for the ASD group were as follows. First, all the children with ASD had an IQ score of 70 or above. This IQ-related criterion was required to ensure that they could understand the test items and instructions and so that any identified differences in performance reflected autistic symptomatology rather than general intellectual functioning. The intelligence test scores were obtained from the children’s school records. In the three countries of interest, a child’s IQ is normally determined by a clinical psychologist or school psychologist using the Arabic version of the Wechsler Intelligence Scale for Children, Third Edition (WISC-III; 1991). Second, they all met the diagnostic criteria for level 1 of the ASD severity scale included within the DSM-5, indicating that they had mild symptoms and required support [49]. Third, all their scores on the Michigan Autism Spectrum Questionnaire (MASQ) [50] were 22 or above, indicating high-functioning performance. Moreover, they all scored ≥ 71 on the Gilliam Autism Rating Scale—Third Edition (GARS-3) [51], indicating that an ASD diagnosis is very likely.

The exclusion criterion for the ASD group was the presence of comorbid affective and/or behavioural symptoms (e.g., intractable epilepsy, severe self-injury, aggression, uncorrected hearing loss, or visual impairment precluding participation). The absence of such comorbidities was verified by the researcher through interviewing the parents and reviewing the children’s medical records.

The comparison (non-ASD or TD) group comprised 30 TD children (age range: 6–12 years; mean age = 9.06 years) recruited from mainstream primary schools. The exclusion criteria were (i) a history of any psychiatric, neurological, or developmental disorder, (ii) a family history of ASD, (iii) a need to regularly use any psychotropic medication, (iv) a physical disability that hinders motor performance, and/or (v) undergoing physical therapy to address motor issues. One obstacle to recruiting a sufficient number of TD participants concerns parents’ reluctance to permit their children to participate in research. This reluctance could indicate that parents are concerned about their children’s assessment results. Data collection can prove particularly challenging in the Arab context due to recognised delays in certain developmental skills and the potential for stigmatisation.

All the participants (i.e., with and without ASD) were group-wise matched according to their gender, chronological age, handedness (rated using the Edinburgh Handedness Inventory (EHI) [52], non-verbal IQ (assessed using Raven’s Coloured Progressive Matrices (RCPM) [53]), and parental education level (see Table 1). The results of the group-wise analyses revealed no significant between-group differences (*p* < 0.05) with regard to any factor relevant to the comparison outcome variables.

### 2.3. Instruments

This study relied on two types of instruments, namely screening questionnaires, and assessment measures.

#### 2.3.1. Screening Questionnaires

The following three screening questionnaires were used to verify the participant’s clinical diagnosis and to determine the severity of their autistic symptoms. The first two measures were completed by at least one parent of each participant with ASD, while the third measure was used by the researcher to independently assess the severity of the ASD symptoms.

1. Gilliam Autism Rating Scale—Third Edition (GARS-3) [51]: After obtaining written permission from the publisher (Pro-Ed), the researcher had previously translated the GARS-3 and made certain cultural adaptations to facilitate its use in the Gulf region. The GARS-3 is a norm-referenced, standardised informant rating scale designed to identify and rate the severity of autism symptomatology in individuals. The GARS-3 items correspond to the diagnostic criteria for ASD set out in the DSM-5 [54]. It consists of 56 Likert-type items that comprise six subtests: restricted/repetitive behaviours (RB; 13 items), social interaction (SI; 14 items), social communication (SC; nine items), emotional responses (ER; eight items), cognitive style (CS; seven items), and maladaptive speech (MS; seven items). The summation of the subscales’ scaled scores yields the composite autism index, which is also reported in terms of the standard score, percentile rank, severity level, and probability of ASD. Two autism indices (four or six) can be formed, depending on whether or not the individual is mute. Higher scaled scores indicate increasingly severe autistic symptoms. Caregivers require approximately 5–10 min to complete the measure.
2. Michigan Autism Spectrum Questionnaire (MASQ) [50]: The MASQ is based on the clinical characteristics that may be suggestive of Asperger’s syndrome (AS) or high-functioning ASD (HFA). It includes ten items representing two main functional areas: the quality of the social interaction patterns and the style of both the content and form of communication. The items are rated on a four-point (0–3) scale, with their sum yielding the total score (maximum 30). A cut-off score ≥ 22 is recommended to screen for individuals with HFA or AS. Cut-off scores between 14 and 21 are predictive of ASD or pervasive developmental disorder-not otherwise specified (PDD-NOS), while scores < 14 are predictive of other psychiatric disorders.
3. The Clinician-Rated Severity of Autism Spectrum and Social Communication Disorders (CRSASSC) [49]: This two-item scale is used to assess the severity of an individual’s autistic symptoms and his/her level of functioning based on the amount of support required due to challenges associated with the social and communication (SC) domain and the restricted interests and repetitive behaviours (RRB) domain, respectively. Each domain is rated on a four-point Likert scale consistent with the DSM-5 diagnostic criteria: 0 (none), 1 (requiring support), 2 (requiring substantial support), or 3 (requiring very substantial support). The clinical criteria may also help to assign a specific functional level to an individual: mild (level 1), moderate (level 2), or severe (level 3). The level of severity for each item should be independently reported, and a combined overall severity score should not be calculated.

#### 2.3.2. Assessment Measures

There are a number of different tools available to assist clinicians and researchers with the evaluation and measurement of different aspects of motor skills development and their contribution to motor performance [55,56]. This study used the following measure.

Bruininks-Oseretsky Test of Motor Proficiency, Second Edition (BOT-2; [48]): The BOT-2 is a standardised, norm-referenced test of motor proficiency used to assess the gross and fine motor skills involved in engaging, goal-directed activities among children aged 4–21 years. It includes eight subtests: fine motor precision, fine motor integration, manual dexterity, bilateral coordination, balance, running speed and agility, and upper-limb coordination and strength. The BOT-2 results in four composite scores and one comprehensive measure of overall motor proficiency. The different administration options include the complete form, the short form, selected composites, and selected subtests. The short form (14 items), which was used in this study, takes 15–20 min to administer, with another five minutes being required to tape off the running course. Liu et al. (2015) noted that it is most efficient for practitioners to evaluate a child using the short form first [56]. The short-form BOT-2 consists of 14 items covering eight subtests and including the widest possible range of abilities to produce sufficiently reliable scores (see Table 2). It is a quick and user-friendly screening tool that provides a single overall motor proficiency score. The total composite score is reported as the standardised score (mean = 50.0, standard deviation = 10.0).

The BOT-2 SF exhibited strong psychometric properties. Its high internal consistency was determined using the stratified alpha method with regard to each composite and the split-half method with regard to each subtest [48]. The alpha value for the total motor composites was found to equal 0.93, while the test-retest correlations were found to be >0.80. Further, the interrater reliability correlations were noted to range between 0.92 and 0.98 [48].

In terms of the validity of the measure, the internal structure of the BOT-2 has been examined using the correlations among the subtest scale scores and the composite scores [57]. The validity of the BOT-2 was also established using a confirmatory factor analysis [58]. The BOT-2 was further determined to be correlated with other measures of motor performance (Bruininks and Bruininks, 2005 [48]. Moreover, the BOT-2 SF was reported to exhibit high sensitivity (84%) but poor specificity (42.9%) with 76.5% accuracy among 153 neurotypical children aged 8–11 years [59]. As per the test manual, the BOT-2 is noted to be able to differentiate between children in different clinical groups, including an ASD group, and children in the normative group.

### 2.4. Procedures

All the involved procedures were carried out in accordance with the ethical standards of the Queensland University of Technology, the research ethics committee of which approved the study protocol. The children involved in this project participated with the full written informed consent of their parents and legal guardians after the study procedures were explained; however, it was also important that the child participants were asked to give their verbal assent to participate. The parents of children with ASD provided information about the severity of their child’s autism by answering the questions on the GARS-3 and MASQ. In addition, the children with ASD underwent an assessment based on the CRSASSC scale that was conducted by the researcher in order to determine the level of severity. Each child was individually evaluated according to the BOT-2 by the researcher in a noise- and distraction-free room at their school. The verbal instructions given were adapted to fit each child’s level of language ability. The instructions for the test were also accompanied by photos of a child performing the main components of the test. All scores were recorded and converted into standardised scores according to the procedure described in the test manual. The assessment sessions were scheduled according to the times suggested by the children’s parents and teachers. The children were all given adequate praise throughout the test. At the start of the testing session, the children were informed that they could cease to participate at any time and for any reason.

### 2.5. Analysis

All the data analyses in the present study were performed using the Statistical Package for the Social Sciences (SPSS) version 23.0 (IBM, Chicago, IL, USA). A one-sample z-test was performed to determine whether the mean of the motor scores of the ASD group statistically differed from the corresponding normative mean derived from the standardisation sample, as available in the BOT-2 manual. Further, the BOT-2 cut-off score (40 or less) was used to calculate the proportions of the actual clinical scores (i.e., the proportion of participants who scored below the clinical cut-off point).

Descriptive statistics were calculated concerning the raw scores for all the BOT-2 subtests. The overall differences between the raw scores of the children with ASD on the BOT-2 and those of the typically developing children were analysed by means of an independent samples *t*-test.

Generally speaking, when performing multiple comparisons using the same data set, the risk of a type I error occurring increases. To overcome this problem, the probabilities were not maintained at the chosen level (0.05), while a Bonferroni adjustment was made to control the overall type I error rate. When performing a Bonferroni adjustment, the alpha is estimated by taking the desired alpha level (0.05) and then dividing it by the number of comparisons being conducted. In the present study, for the multiple comparison analysis of the eight BOT-2 subtests, the significance level was set at *p* = 0.006.

The effect sizes were expressed using Cohen’s d [60]. According to Cohen (1988), the magnitude of the effect size can be categorised into one of three graded levels, namely small (0.2–0.5), medium (0.5–0.8) or large (>0.8) [60].

Further, the effect of the age variable on the participants’ motor performance was investigated using a regression analysis, with age being considered the predictor variable and the total raw score for each child’s motor performance being considered the outcome of interest. The level of statistical significance was set as *p* < 0.01 in order to avoid type I errors.

For linear regression, the effect size was calculated using Cohen’s ƒ2. According to Cohen’s guidelines (1988) concerning the interpretation of the magnitude of the R-squared value, the values of Cohen’s ƒ2 = 0.02, 0.13 and 0.26 are considered to be small, medium, and large effect size, respectively. The equation to compute Cohen’s effect size is (ƒ2 = R2/1 – R2) [60].

## 3. Results

The results of this study are presented in three stages according to the sequence of the research objectives:

### 3.1. The Proportion of Children with ASD Who Exhibit Motor Impairments

The BOT-2 standardized test was used in this study in order to evaluate motor proficiency in the child participants. Lower scores on the BOT-2 items are considered to be indicative of lower motor performance. On the short form of the BOT-2, the subtests are administered to find the raw scores. Each raw score for the subtests was then converted into a point score using the graded scale provided. The point scores are summed to give the total point score (max = 88), which is in turn converted into the standard score considering age and sex (ranging from 20 to 80), with a mean of 50 (SD = 10). A total composite standard score of 40 or less (one standard deviation below) is classified as indicative of motor impairment. This total motor composite can be reported as a categorical variable with five descriptive categories: well above average “≥70”, above average “60–69”, average “41–59”, below average “31–40”, and well below average “≤30”. Based on this process, the overall score, which is the sum of the eight subscales, was compared with the BOT-2 norms. Table 3 presents descriptive statistics concerning the total BOT-2 motor composite scores of the children with ASD as well as the results of the z-test comparing their overall motor performance to the performance of the normative test sample.

As shown in Table 3, the mean BOT-2 score of the ASD group for the total motor composite (M = 31.90, SD = 5.03, *p* < 0.001) statistically deviated from the norm with a large effect size; thereby, indicating the presence of motor impairment within the overall level of motor proficiency. In light of the cut-off score set out in the BOT-2 guidelines, 88% of participants with ASD scored below the normal threshold when compared with the normative BOT-2 data. Based on the descriptive standard scoring categories detailed in the BOT-2 guidelines, the children with ASD were classified as displaying a motor performance deficit that fell within the below-average range.

### 3.2. Comparing the ASD and TD Children Based on the BOT-2 Test Raw Scores

Table 4 shows the participants’ mean raw scores and standard deviations on the eight BOT-2 subtests, namely fine motor precision, fine motor integration, manual dexterity, upper-limb coordination, bilateral coordination, balance, running speed and agility, and strength, as well as the total motor composite. It therefore indicates the differences identified between the children in the ASD group and the typically developing children. The highest point score for the test is 88. Higher scores on the BOT-2 items are indicative of higher motor performance, and vice versa.

The independent samples *t*-test found significant differences in terms of all the BOT-2 short-form subtests and the total score when comparing the children with ASD and the typically developing children. All the differences are significant at the *p* < 0.001 level, with the size effects on the eight BOT-2 subtests ranging from 1.16 to 2.66 and the total motor composite equalling 2.71 (Cohen’s *d*). This indicates that the children with ASD performed more poorly on the BOT-2 items than the TD controls. The performance on the eight motor subtests was variable. The children with ASD scored their lowest point scores on the strength element of all the subtests (Cohen’s *d* = 2.66), while Fine Motor Integration was associated with their highest point scores (Cohen’s *d* = 1.16).

### 3.3. Relationship between Motor Performance and Age

The relationship between the ASD participants’ age and their overall motor performance was examined using linear regression analyses. The results indicated the significant main effect of age, which suggested that the children with ASD exhibited age-related gains in terms of their overall motor performance. Indeed, positive correlation was noted between their overall motor performance and their age (*β* = 0.52, *p* ≤ 0.001, *R^2^* = 0.26, Cohen’s ƒ^2^ = 0.36). This correlation pattern indicated that the children with ASD exhibited fewer deficits during the performance of motor tasks as they aged.

## 4. Discussion

The results relevant to each aim of the study will be discussed separately in the following subsections

### 4.1. Prevalence of Children with ASD Who Exhibited Clinically Significant Motor Abnormalities

Our first objective was to identify the prevalence of children with ASD who exhibited clinically significant motor abnormalities. The results indicated that the majority of the ASD sample fell outside the normal range in terms of motor performance. Indeed, the prevalence of children with ASD who exhibited clinically significant motor performance problems was found to be 88%. These children had a score of 40 or less on the total composite, which indicates deficits in their motor skills.

These results regarding the motor impairments seen in children with ASD are consistent with our expectations, since they reflect the motor abnormalities seen in those with autism. Additionally, such findings regarding deficient motor functioning in children with ASD are in line with the findings of several prior studies that relied on a similar measure to the BOT-2 [8,17,29,61,62,63]. The mean standard score for the overall motor composite in these previous studies ranged from 33.0 to 39.6, indicating the existence of motor impairments among children with ASD. A similar pattern of results was also found in the clinical sample of 45 individuals with autism aged 4 –21 years described in the BOT-2 manual (M = 37.0; SD = 8.4) [48]. The present results are also in line with those of other studies that used different measures [6,11]. However, our results contrast with those found in a study by Hilton et al. (2014) that used the BOT-2 but did not identify a significant deficit in the motor skills of individuals with ASD [64]. However, the small sample size involved in Hilton et al.’s (2014) study should be considered, since they only investigated seven children with ASD.

The motor disruptions seen in children with ASD may be attributed to the increase in the total brain volume seen in such children, as well as to certain affected brain regions that are regularly suspected to be involved in the neural underpinnings of autism, including the cerebellum, basal ganglia, brain stem functions, and alterations in the frontostriatal and frontocerebellar pathways [10,65]. However, the brain mechanisms underlying the motor disruptions observed in those with autism are not conclusive, meaning that they warrant further investigation.

Environmental factors may also contribute to the poor motor skills performance exhibited by those with ASD. An individual’s motor skills development stems from the dynamic relationship that exists between organismic and environmental factors; hence, any changes in such properties will influence the acquisition of motor skills [66]. A study by Maksoud (2016) indicated that certain environmental factors might explain the poor performance exhibited by Egyptian children with Down’s syndrome in relation to the BOT-2 instrument [67]. These factors included low enhancement and poor support from the parent(s), as well as limited opportunities to practice during the early years of their lives [67]. A proposition drawn from these findings is that these factors may also be applicable to children with ASD.

It is important to note, however, that the prevalence of motor performance abnormalities identified in the context of our research was high when compared to the prevalence reported in other studies [17,61,62,63]. It is possible that the high frequency of motor performance abnormalities seen in the children with ASD in the present study could be attributed to several factors largely related to the local cultural context.

First, low levels of physical activity can contribute to reducing the motor proficiency of children with ASD. The relationship between an individual’s motor proficiency level and his/her level of physical activity was confirmed by Wrotniak, Epstein, Dorn, Jones, and Kondilis (2006) [68]. The majority of families in the Gulf region depend on domestic servants to help care for their children and to do the housework [69]. These domestic servants also typically assist the children with numerous tasks associated with daily living, especially in the case of children who have been diagnosed with a disability. This assistance could involve, for example, buttoning up clothes, zipping up zippers, tying shoes, bathing, feeding, cleaning teeth, and combing hair. Hence, it is possible that the children might come to overly rely on the domestic servants to help them perform everyday activities. This could in turn have a negative impact on the physical activity levels of the children, and therefore adversely affect their motor development.

Second, a lack of opportunities to engage in physical activity/practice responding to physical stimuli could stem from various familial factors. The negative relationship between motor development and insufficient physical activity has been confirmed in the literature [70,71]. The parents of children with ASD in the Arab Gulf region tend toward an overprotective parenting style, and they often exhibit excessive concern about their children’s safety when outdoors. This parenting style is likely to have contributed to the children with ASD being less physically active than is ideal and, relatedly, to their insufficient motor skills proficiency [72].

Additionally, children living in the Gulf region tend to only rarely engage in physical activities out in the natural environment. Prior research evidence has suggested that the physical environment that we inhabit can offer both opportunities and barriers in terms of engaging in physical activities [70]. For example, countries in the Gulf region frequently experience very high temperatures during the daytime [73]. Therefore, it is rarely considered safe or appropriate for children to engage in outdoor activities during that time/in such conditions. Children with ASD are particularly prone to being prevented for participating in outdoor activities due to both the perceived inconvenience of them doing so and the associated security concerns. This lack of participation may affect the development of the children’s gross motor skills.

Finally, children with ASD typically lack exercise partners of the same age and are frequently isolated from their peers, which means that they generally lack opportunities to engage in social interactions. Unfortunately, children with ASD are particularly susceptible to social exclusion due to both the stigma experienced by their families (i.e., feelings of shame about their child’s condition) and the rejection and lack of acceptance shown by their peers (i.e., evasion and discrimination). Some families attempt to keep their children’s condition a secret, and hence prevent their children from attending social events and engaging in social interactions with their peers. It has been reported that the lack of a peer exercise partner represents one of the key barriers to physical activity experienced by children with ASD [74]. Such non-participation in peer activities may limit children’s opportunities to develop their motor proficiency.

### 4.2. Distinct Nature of Motor Performance Exhibited by Children with ASD and Typically Developing Children

This study also sought to examine the differences between the motor performance, as assessed using the BOT-2, of children with ASD and typically developing children. The results demonstrated that the children with ASD exhibited weaker motor skills performance than the typically developing children, which indicated deficits in their motor proficiency. The results, therefore, support the findings of prior studies concerning motor behaviours, which indicated a general impairment of motor functioning in individuals with ASD [10,11]. The results contribute to our understanding of definitive areas of motor dysfunction among children with ASD and their long-term developmental consequences. Further, the results add weight to previous insights intended to assist caregivers and therapists in addressing such issues. Overall, the results provide further evidence of the need to reconceptualise ASD to include motor delays and deficits.

When considering all eight subscales used to evaluate the overall motor proficiency of the children with ASD, it appears that the identified motor deficits manifested with different degrees of severity. For instance, fine motor precision tasks require precise control over finger and hand movements. Our results indicated that the children with ASD experienced difficulties drawing lines through paths and folding paper. This finding is in accordance with the notion that individuals with ASD experience difficulties completing tasks that require the planning and sequencing of movement [75]. As some children with ASD also exhibit hypotonia, it is likely that the hypotonia in their hands results in severe fine-motor and graph-motor delays due to an inability to manipulate objects and control a pencil [76]. Such difficulties may lead to problems during many activities associated with daily living, including buttoning clothes and writing.

Fine motor integration skills require the capacity to integrate visual stimuli with motor control. This kind of integration is commonly referred to as “visual motor integration”. Our results indicated that the children with ASD were generally unable to perform the hand-eye coordination tasks. This finding is in line with prior evidence that some individuals with ASD exhibit underlying deficits in visual-motor integration [77]. Such deficits may stem from sensory problems since hand-eye coordination requires the assimilation of several types of sensory inputs to direct movement toward the target [78]. Moreover, children with ASD demonstrate delayed manipulation skills, which are also important for activities such as writing and fastening clothing [79]. These fine motor integration skills tend to be affected when an individual with ASD experiences a tactile perception problem, especially when tactile defensiveness is present. Ayres noted that hyper-responsivity to tactile stimuli may render it difficult for younger children to develop in-hand manipulation, fine motor skills, and hand-eye coordination [80]. It has been established that children with ASD often exhibit difficulties with tactile awareness, which may impede their fine motor skills. Thus, it has a bearing on their ability to develop fine motor skills.

Our findings also revealed the impaired performance of the children with ASD on the manual dexterity subtest, which is in line with the results of prior studies [11,14,81,82]. Deficits in manual coordination may occur due to the poor hand-eye coordination typically seen in children with ASD, since good hand-eye coordination allows individuals to pick up small objects and precisely manipulate them. Further, perception-action coupling is crucial to the production of purposeful, coherent movements [14]. However, prior evidence has suggested that such manual coordination deficits may be related to the use of a dual-scored task that involves both spatial accuracy and age-related temporal parameters [14]. Interestingly, a recent study indicated that the worst results for children with ASD were seen in relation to the fine motor skills component, while the gross motor skills component gave rise to the best results [83]. During the indicative qualitative evaluation, the researchers found more children with ASD to be able to manage the manual tasks when provided with unlimited time and support [83]. In contrast, the majority of children with ASD could not manage the gross motor tasks at all [83].

The bilateral coordination subtest concerns the capacity to use both sides of the body in a controlled and organized manner to accomplish a functional task [79]. The results indicated that the children with ASD experienced problems when attempting movements involving both sides of the body. This finding is consistent with the fact that individuals with ASD are typically characterised by poor bilateral coordination [84]. Further, Staples and Reid found that children with ASD experienced difficulty coordinating both sides of their body, or both arms and legs, while performing motor tasks [82]. Bilateral coordination problems may be associated with vestibular dysfunction, as when the vestibular system is unable to adequately integrate information, it can contribute to poor bilateral coordination. Moreover, the vestibular system allows the two sides of the body to communicate with each other at the level of the brain stem via the vestibular nuclei [85].

The balance subtest evaluates the motor control skills integral to an individual’s posture when standing. Balance is necessary for both movement and stillness, which is why it is sometimes referred to as postural control [86,87]. The balance subtest consists of movement activities that measure the stability of the trunk support as well as stasis and movement. Our results suggested that the balance skills of the children with ASD were significantly impaired when compared with the typically developing children, which is in line with the findings of previous studies [10,11,14,81]. However, Ament et al. found that deficits in the balance skills of children with ASD were only demonstrated during static balance tasks and not during dynamic balance tasks [88]. Further, the presence of balance deficits in children with ASD is often interpreted as indicating a deficit in the integration of information obtained from the visual, proprioception and vestibular afferent systems [10,89]. The maintenance of successful postural stability during static balance postures is thought to require the integration of vestibular, somatosensory, and visual inputs. If one of these inputs is impaired or disrupted, then both simple and complex motor tasks prove more difficult [19].

The running speed and agility subtest includes activities that require both speed and agility. Hopping is defined as the elevation of the body off the ground and the subsequent landing on a single foot [90]. It appears to be an extension of the ability to balance while standing on one leg. This type of balance is referred to as “dynamic balance”. Our results indicated that the children with ASD experienced difficulty in hopping on one foot when compared with the typically developing children. This is in accordance with prior clinical observations that many autistic children experience great difficulty when hopping [91,92]. For instance, Noterdaeme et al. found that children with ASD experienced difficulties performing motor skills that require standing and hopping on one foot for a predetermined period of time when compared with typically developing children [93]. A similar result was found by Pusponegoro et al., who noted that only five out of 40 (12.5%) children with ASD could hop on one foot without falling over [92]. Comparable to the situation with balance and bilateral coordination, a possible contributing factor to the impairment seen in children with ASD is the vestibular problems they experience. Hopping requires the ability to balance on either foot, which relies heavily on a sensitive vestibular system and motor coordination [94]. The vestibular system also plays an important role in controlling the mutual interaction of sensory inputs and motor outputs [95]. As the ASD population typically exhibits an impairment in vestibular processing that results in poor balance, this impairment may impact the ability of a child with ASD to hop.

The upper-limb coordination (ULC) subtest consists of activities designed to measure visual tracking with coordinated arm and hand movements. Our results revealed that the children with ASD exhibited ULC difficulties, which is consistent with prior evidence that individuals with ASD exhibit poor ULC during visuomotor activities [96]. The results also indicated that the use of both hands to drop and catch a ball proved difficult for the children with ASD [14]. It appears, therefore, that children with ASD experience difficulty completing tasks involving both hands. When attempting to catch a ball with two hands, their arm movements are often poorly coordinated [97,98]. These deficits may be attributed to insufficient information processing [99]. As activities involving ball skills are often social in nature, such motor impairments may result in the exclusion of children with ASD from games, which may disrupt their psychosocial development [98,100].

The strength subtest is intended to assess the trunk flexor muscles and the upper and lower extremity strength. It involves activities such as push-ups and sit-ups. Our results indicated that the children with ASD showed an insufficient level of strength during such activities. They were able to complete fewer push-up and sit-up repetitions than the typically developing children. This is in line with the results obtained by Pan (2014), who found individuals with ASD to be able to complete fewer push-ups and sit-ups than age-matched peers without ASD [29]. The inability to master the push-up skill may indicate weakness in an individual’s general upper body condition. Further, difficulty performing sit-ups may result from relatively low middle-body strength [101]. One explanation for the poor performance of the children with ASD in relation to strength skills could be their limited general body strength. Such activities require a great deal of muscle strength, which is also necessary for many aspects of movement and motor control, including postural control, balance, and coordination [102].

### 4.3. The Relationship between Age and Motor Performance

The final aim of this study was to determine whether the chronological age of the children with ASD affected their overall motor performance. Ultimately, it was determined that age did impact the motor performance of the children with ASD. The total score for motor performance was positively related to the age of the children with ASD. In general, the present results indicate that children with ASD demonstrated fewer deficits during motor tasks with age. This finding is consistent with the general pattern of the developmental acquisition of motor skills with age [43,48]. Relatively few studies have examined age differences in terms of motor performance in those with ASD, although the current results are in line with those of a study by Ming et al. (2007), who found that older children with ASD tend to perform better in relation to the motor skills than younger children with ASD [16]. However, the present results stand in contrast to the results of longitudinal work indicating that these motor deficits remain relatively stable over time in children with ASD [46]. The identified age-related gains could be due to the natural maturity of motor skills or the result of therapeutic interventions [16]. Structural environmental changes may promote motor development in children with ASD. However, the children with ASD in the context in which this study was conducted, whether they exhibited motor deficits or not, were not expected to receive any services to assist with their motor development. Hence, the results of the present study support the hypothesis that the development of fine and gross motor skills is characterized by change rather than stability.

## 5. Conclusions

The present study outlined the prevalence of motor impairments in children with ASD, as well as the nature of the motor abnormalities seen in school-aged children with ASD when compared to typically developing children, through the use of a standardized test so as to facilitate a better understanding of the motor profiles associated with ASD. An additional focus of the study was the exploration of the relationship between motor performance ability and age. Based on the impairment cut-off detailed in the BOT-2 guidelines, the majority of children with ASD in our sample showed mild to extremely negative deviation from population norms, and therefore were classified as exhibiting a motor performance deficit. This study also found evidence that the children with ASD performed significantly poorer than the typically developing children in relation to all the items of the BOT-2. In addition, the study demonstrated the existence of a positive relationship between age and motor performance ability, which indicated that motor skills may improve as children grow older.

The overall findings of this study served to reinforce and extend the results of prior studies concerning motor abnormalities in those with ASD. In particular, the present findings contributed significantly to our understanding of such motor characteristics in non-Western individuals diagnosed with ASD.

A strength of this study is related to the use of a standardised assessment of motor impairment (i.e., the BOT-2), which is one of the most commonly applied means of evaluating and discriminating motor function in children. However, only the short form of the chosen motor proficiency test (SFBOT-2) was used in this study, which limited our interpretation of the subcategories. However, the use of the SFBOT-2 can be justified based on the findings of our unpublished pilot study, which revealed both the difficulty and the high time requirements associated with applying the full version of the test. Additionally, a number of comorbid psychiatric disorders, including anxiety, could potentially impact children’s performance during a motor task [103,104]. However, the results of this study do not contribute to the ongoing discussion regarding the effect of anxiety on test performance. Thus, future studies could examine the possible relationship between anxiety levels and performance levels during motor tasks. Although the severity of the ASD symptoms has been found to be related to the severity of the experienced motor problems [8,105,106,107], examining this relationship in more detail is beyond the scope of the present study. It does, however, represent an interesting avenue for future studies to explore.

Another limitation of the present study is related to the cross-sectional methodological approach chosen to explore the impact of chronological age on the motor difficulties exhibited by children with ASD, which limited our ability to interpret the findings from a developmental perspective. Future studies should employ a longitudinal design so as to be better able to investigate the developmental trajectories of motor impairments in children with ASD and to provide us with further insights into the mechanisms that underlie such motor disruptions. The final limitation of this study concerned the study sample. Only children with ASD who exhibited an average or above-average IQ were included in the study. Therefore, the findings are limited to that group, and hence are not representative of individuals with ASD who exhibit lower cognitive function. Additionally, the fact that the sample of typically developing children included in the study was relatively small should be considered. Indeed, the typically developing participants in this study may not be representative of the population as a whole.

Despite the above-mentioned limitations, the findings of the present study have a number of important implications for practice. First, the results reported here suggested that motor abnormalities represent a prevalent feature of ASD. However, such problems do not typically form part of the assessments for ASD nor are they typically included in intervention programs. Therefore, the inclusion of a motor assessment in the evaluation protocols associated with ASD should prove highly useful. Second, although motor deficits are present from very early on in the development of children with ASD and may actually reflect an underlying brain deficit, they do not generally represent a key focus for either parents or professionals. It is recommended that motor functioning should be considered during the clinical assessment and management of ASD, and it should not be ignored during early interventions. Further, it is recommended that additional research be undertaken in this regard so as to ensure greater clinical recognition of motor dysfunctions among children with ASD.

Moreover, the wide-ranging implications of poor motor performance on the part of individuals with ASD can extend beyond the motor domain and into the educational, social, cognitive, and behavioural domains. Hence, motor functioning represents another area that we should be mindful of in the clinical context. The identification of motor abnormalities could be a potentially valuable marker of the degree and the nature of the other defining clinical features of ASD. Finally, the results of the present study highlighted the need for increased efforts to improve the clinical surveillance process in order to better identify potential developmental delays or deficits in terms of motor performance, as well as to implement intervention strategies intended to address motor problems, in children with ASD. Early identification and proper intervention with regard to motor problems in children with ASD may lead to such children experiencing improved self-confidence and self-esteem, increased performance quality, and enhanced occupational engagement and social participation.

## Figures and Tables

**Table 1:** Demographic and Clinical Characteristics of the Participants.

Comparison Characteristics		Target Group (*N* = 119) Children with ASD	Control Group (*N* = 30) TD Children	t/χ^2^	*p*	ES
**Continuous variables**						
Age (years)		8.72 (1.96) *	9.06 (1.42) *	1.05	0.71	0.21
Non-verbal IQ		29.76 (1.92) *	29.80 (2.64) *	0.69	0.060	0.17
**Categorical variables**						
Gender	Male	95 (79.8%)	24 (80.0%)	0.00	0.98	-
	Female	24 (20.2%)	6 (20.0%)			
Handedness	Right	104 (87.4%)	23 (76.7%)	2.191	0.14	-
	Lift	15 (12.6%)	7(23.3%)			
Father’s education level	Secondary	67 (56.3%)	16 (53.3%)	0.09	0.77	-
	College degree	52 (43.7%)	14 (46.7%)			
Mother’s education level	Secondary	78 (65.5%)	20 (66.7%)	0.01	0.91	-
	College degree	41(34.5%)	10 (33.3%)			

Note: * Mean (SD) non-verbal IQ (Raven’s raw score range 0–36). The continuous variables are summarised as the mean and standard deviation using *t*-tests, while the categorical variables are summarised as the count and percentage using the chi-square χ^2^. ES = effect size, which is calculated using Cohen’s d.

**Table 2:** Short-Form Items.

The BOT-2 Subtest	Item	Assessment
**Subtest 1** *Fine Motor Precision*	Drawing Lines Through Paths	# of Errors
	Folding Paper	# of Errors
**Subtest 2** *Fine Motor Integration*	Copying a Square	# of Errors
	Copying a Star	# of Errors
**Subtest 3** *Manual Dexterity*	Transferring Pennies	# of Pennies in 15 s
**Subtest 4** *Bilateral Coordination*	Jumping in Place-Same Sides Synchronized	Repetitions
	Tapping Feet and Fingers-Same Sides Synchronized	Repetitions
**Subtest 5** *Balance*	walking Forward on a Line	Steps
	Standing on One Leg on a Balance Beam-Eyes Open	Time
**Subtest 6** *Running Speed Agility*	One-Legged Stationary Hop	# of Hops in 15 s
**Subtest 7** *Upper-Limb Coordination*	Dropping and Catching a Ball-Both Hands	Catches
	Dropping a Ball-Alternating Hands	Dribbles
**Subtest 8** *Strength*	Full Push-Ups	# Performed in 30 s
	Sit-Ups	# Performed in 30 s

Note: (#) The hash symbol expresses the number of errors made during task completion and the number of tasks completed.

**Table 3:** Descriptive Statistics and Z-Test Results Concerning the Total Motor Composite.

BOT-2	ASD Sample (*N* = 119)					95% Confidence Interval for the Mean		One-Sample Z-Test		
Standard score for the total motor composite *	**Min**	**Max**	**M**	**SD**	**SE**	**Lower**	**Upper**	**Z**	***p***	**d**
	47.0	85.0	31.90	5.03	0.46	30.98	32.81	−19.74	<0.001	1.81

Note: * A standard score less than 40 is regarded as the cut-off point for an abnormal motor performance.

**Table 4:** Comparison of the autism spectrum disorder and typically developing groups based on the BOT-2 raw scores and total motor composite.

The BOT-2 Test Scores	ASD Sample (*N* = 119)		TDC Group (*N* = 30)		t-Statistic	*p*	D
	M	SD	M	SD			
Fine Motor Precision	5.38	2.66	10.17	2.10	9.171 ***	<0.001 ***	1.99
Fine Motor Integration	7.43	2.17	9.53	1.36	6.623 ***	<0.001 ***	1.16
Manual Dexterity	3.28	1.62	6.33	1.56	9.322 ***	<0.001 ***	1.92
Bilateral Coordination	4.60	1.68	6.67	0.85	9.489 ***	<0.001 ***	1.55
Balance	5.69	1.10	7.30	0.75	7.563 ***	<0.001 ***	1.71
Running Speed and Agility	3.08	2.03	6.97	1.40	12.267 ***	<0.001 ***	2.23
Upper-Limb Coordination	5.13	2.80	10.20	1.73	12.472 ***	<0.001 ***	2.18
Strength	3.21	2.08	8.97	2.24	13.352 ***	<0.001 ***	2.66
Total Motor Composite	37.79	12.25	66.13	8.24	15.097 ***	<0.001 ***	2.71

Note: *** *p* < 0.001 by independent samples *t*-test. ADS: Autism spectrum disorder without intellectual disability; TDC: typically developing children; BOT-2: Bruininks-Oseretsky Test of Motor Proficiency, Second Edition; d values are effect sizes (ESs) of ASD versus TDC. Bonferroni correction: α  =  0.05/8  = 0.006.
